# Treatment of oral lichen planus with mucoadhesive mycophenolate mofetil patch: A randomized clinical trial

**DOI:** 10.1002/cre2.302

**Published:** 2020-06-26

**Authors:** Negin Samiee, Ali Taghavi Zenuz, Masumeh Mehdipour, Javad Shokri

**Affiliations:** ^1^ Post Graduate Student of Oral and Maxillofacial Medicine, Oral and Maxillofacial Medicine Department Mashhad Dental Faculty Mashhad Iran; ^2^ Department of Oral and Maxillofacial Medicine, School of Dentistry Tabriz University of Medical Sciences Tabriz Iran; ^3^ Department of Oral and Maxillofacial Medicine, School of Dentistry Shahid Beheshti University of Medical Sciences Tehran Iran; ^4^ Department of Pharmaceutics, School of Pharmacy Tabriz University of Medical Sciences Tabriz Iran

**Keywords:** burning, mucoadhesive, mycophenolate mofetil, oral lichen planus, topical, treatment

## Abstract

**Objectives:**

Oral lichen planus (OLP) is a chronic inflammatory disease of unknown etiology which is known as a premalignant disease. A complete cure has not been found for this condition. Mycophenolate mofetil (MMF) is a new drug that seems to be effective in improving OLP lesions. But there are no studies on the efficacy of mucoadhesive form of MMF in ulcerative OLP. Therefore, this study was performed to determine the therapeutic effect of MMF mucoadhesive on OLP lesions.

**Material and methods:**

Twenty‐seven patients with OLP, were enrolled in two groups. All the patients were instructed to place the MMF 2% mucoadhesive on the lesion twice daily for 4 weeks. Lesion size was measured by a sterile digital caulis (mm) and the severity of burning sensation and pain by visual analogue scale (VAS; cm) at baseline and weekly follow‐ups.

**Results:**

There was no significant difference in burning sensation and lesion size at Weeks 1, 2, and 3 in both groups. In Group A, at Week 4, there was significant reduction in pain and burning sensation and lesion size on both sides (*p* = .048, .012). The difference in lesion size on control sides was not significant. In Group B, at Week 4, there was significant reduction in pain and burning sensation and lesion size (*p* = .004). No side effects were reported by the patients.

**Conclusions:**

Based on the results, 2% MMF mucoadhesive was effective in decreasing burning sensation and pain severity and ulcer size of ulcerative OLP and the effect was time‐dependent.

## 1 INTRODUCTION

Lichen planus (LP) is a common inflammatory disease of the skin, mucous membranes, nail, and hair follicles (Nazemi, Esmaeli, Sedaghat, & Mostafa, 2006), in which autoreactive T lymphocytes are directed against basal layer antigens. The lesions can vary from a mild inflammation to destruction of the epithelium with painful wounds (Glick, 2015). Although it is a premalignant disease, a complete cure has not been found for it yet. The most commonly employed and useful agents for treating oral lichen planus (OLP) are systemic and topical corticosteroids (Beigom Taheri et al., 2010). Activated T cells are important in the pathogenesis of LP as indicated by the dermal lymphocytic infiltrate, leading to keratinocyte destruction and lesion formation. Mycophenolate mofetil (MMF) is an immunosuppressive drug which specifically and reversibly inhibits the proliferation of activated T cells. It has also been successfully used in the treatment of graft‐versus‐host disease (GVHD; Frieling, Bonsmann, Schwarz, Luger, & Beissert, 2003).

Over the last few years, MMF has also emerged as an alternative therapeutic regimen for patients affected by other autoimmune vesiculobullous diseases, in order to decrease the dose and side effects of corticosteroids (Iaccarino et al., 2007). It has been successfully used in the treatment of skin diseases, such as LP, and rheumatoid and immunologic diseases (Mutasim, 2004). Frieling et al. (2003) examined the therapeutic potential of 1 g of MMF capsule daily in three patients suffering from disseminated erosive LP. MMF was well tolerated and induced complete remission in two patients and substantial improvement in the third. Cho et al. (2010) used 0.5 g of oral MMF twice daily for 4 weeks and reported an 83% decrease in symptoms of refractory lichen planus. Dalmau et al. (2007) first used oral MMF and cyclosporine followed by 2 g of MMF daily for 2 months and 1.5 g of MMF daily for the next 2 months in a patient with refractory OLP and reported complete remission in the ulcerative sites of it. It is generally a well‐tolerated immunosuppressive agent with a preferred side effect profile compared to other immunosuppressive drugs because it has less nephrotoxic, hepatotoxic, and neurotoxic effects (Orvis et al., 2009). In the previous studies, no relevant short‐term or long‐term side effects were reported by participants, except for minor gastrointestinal disturbances at the beginning of MMF treatment (Frieling et al., 2003); of course, all the side effects are for the systemic prescribed MMF.

Oral mucoadhesive delivery system provides sufficient time for drug absorbance and high concentration of it by increasing drug's contact with the lesion. In addition, since the drug has no effects on healthy tissues around the lesion, it seems to have no side effects on them. Importantly, because of concentration of the drug on the lesion, much lower dose of the drug is used. All of these are the advantages of mucoadhesives over other drug delivery systems, like solutions, gels, ointments, and oral sprays (Shaikh, Raj Singh, Garland, Woolfson, & Donnelly, 2011).

Thus, due to the effectiveness of MMF on LP, with rarely reported side effects and advantages of mucoadhesive drug delivery system; we aimed to evaluate the efficacy of mucoadhesive form of MMF in decreasing symptoms of oral erosive lichen planus lesions in a sample of Iranian patients.

This study was conducted from November 2012 to August 2013.At the time of the study, according to our investigations, there was no report of topical prescription of MMF except for Wohrlab (Wohlrab, Jahn, Plaetzer, Neubert, & Marsch, 2001) which had studied 2% topical MMF in patients with psoriasis and found a significant decrease in symptoms of skin lesions of psoriasis. Due to the similarity of immune system function in pathogenesis of both lesions of lichen planus and psoriasis, and to observe medical ethics, it was decided to use a similar percentage of the drug (2%) in this study.

## 2 MATERIALS AND METHODS

### 2.1 Participants

In this clinical trial, 27 patients, referred to the Department of Oral Medicine, Faculty of Dentistry, Tabriz University of Medical Sciences and evaluated by modified WHO criteria for OLP diagnosis (Van der Meij & Van der Waal, 2003), were included. Initially, it was decided to include only patients with bilateral lesions; but due to the small number of them, patients with unilateral lesions were also included in the study. Finally, 10 patients with bilateral ulcerative lesions of OLP, were enrolled in a double‐blind clinical trial (Group A) and 17 patients with unilateral ulcerative lesion of OLP were enrolled in a before–after clinical trial (Group B).

#### 2.1.1 Inclusion criteria

Patients suffering from erosive OLP with clinical manifestations and histopathological criteria; healthy patients older than 18 years of age.

#### 2.1.2 Exclusion criteria

Received any treatment for the lesions in the past 4 weeks; presence of lichenoid reaction; pregnant or breast feeding women; any systemic or dermal disease or simultaneous use of other drugs affecting the immune system of the patient; incidence of any adverse effects of MMF during the study.

### 2.2 Mucoadhesive production

To produce MMF 2% mucoadhesive patch, we first used proper solvent system to be able to dissolve both the polymers used and the drug homogenously with high volatility so that it can be separated from other materials. In this study, water, ethanol, acetone mixture with 25, 50, and 25% volumetric ratios were used. Acetone was added to the solvent system as an excellent solvent for MMF.

Ingredients were the active drug, two types of light and heavy polyethylene glycols grades 300 and 2,000 (Merck Germany) as thin‐film polymers and Polyvinyl Alcohol grade K90 (BASF Germany), hydroxypropyl methyl cellulose (HPMC) methylcellulose grade K4M (Colorcon England) as adhesive polymers and diethyl phthalate as a plasticizer.

**FIGURE 1:**
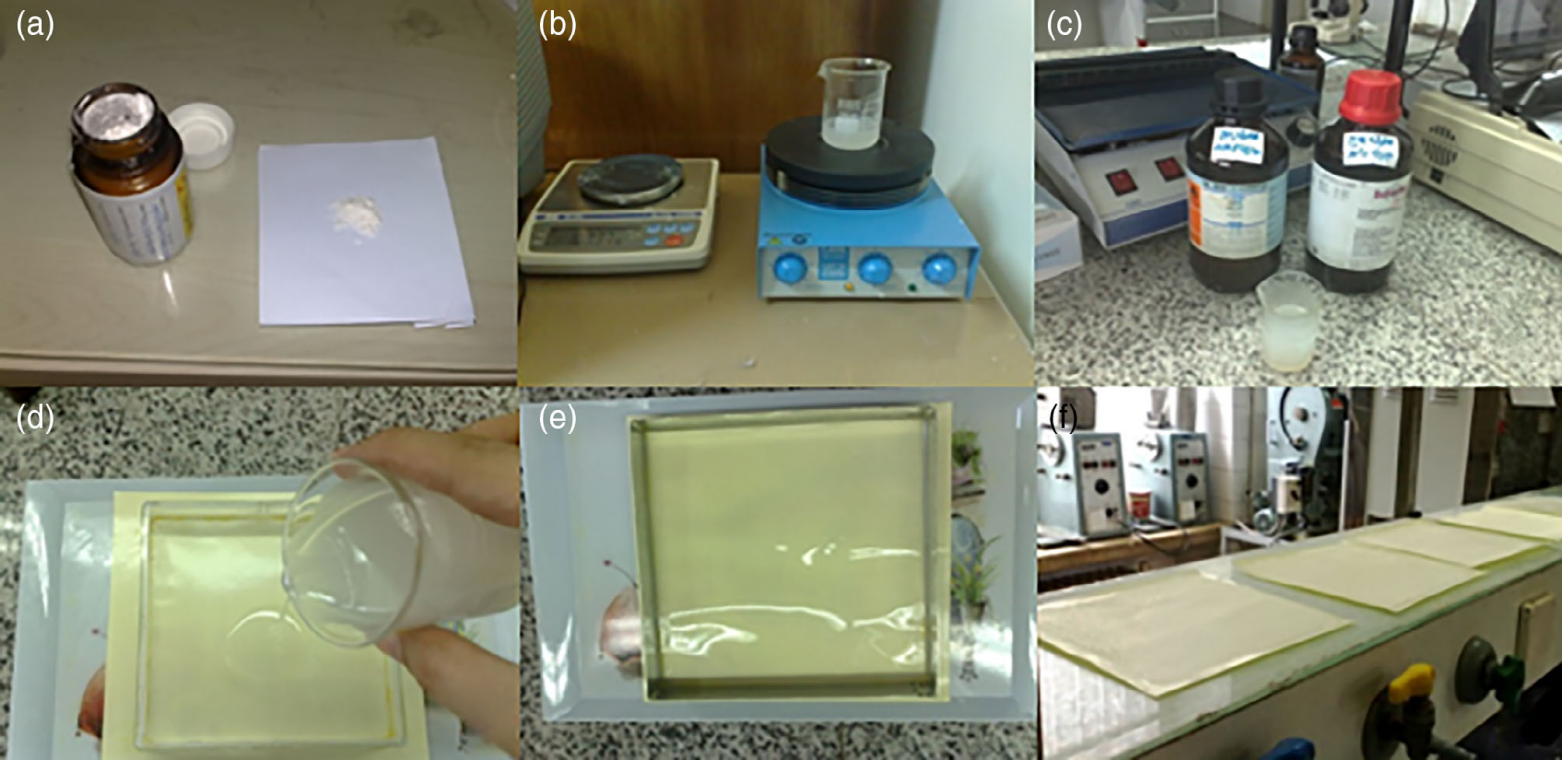
MMF mucoadhesive production. MMF, mycophenolate mofetil

To prepare the adhesive patch, all the formulation components were first inserted into the mixture of the solvents in the beaker and the lid was completely blocked with paraffin to prevent the evaporation of the solvent. The resulting mixture was stirred for 24 hr in a magnetic stirrer until the components were completely dissolved and the viscous and uniform solution was obtained (Figure 1a–c) and then spread over a silicone flat plate to let the solvents evaporate. A transparent, very thin polymeric layer containing the drug remained on the silicon plate. It was used as MMF 2% mucoadhesive patch (Figure 1d–f), kept in moisture‐resistant containers to prevent drying of the patch. All components and polymers used in this formulation are soluble in water, so it dissolves slowly in saliva and releases the active drug, when used intraorally. The thicker the layer, the longer it will take to completely dissolve. So, in this study, the thickness was adjusted to be completely resolved within 2–3 hr.

### 2.3 Interventions, randomization, and blinding

**FIGURE 2:**
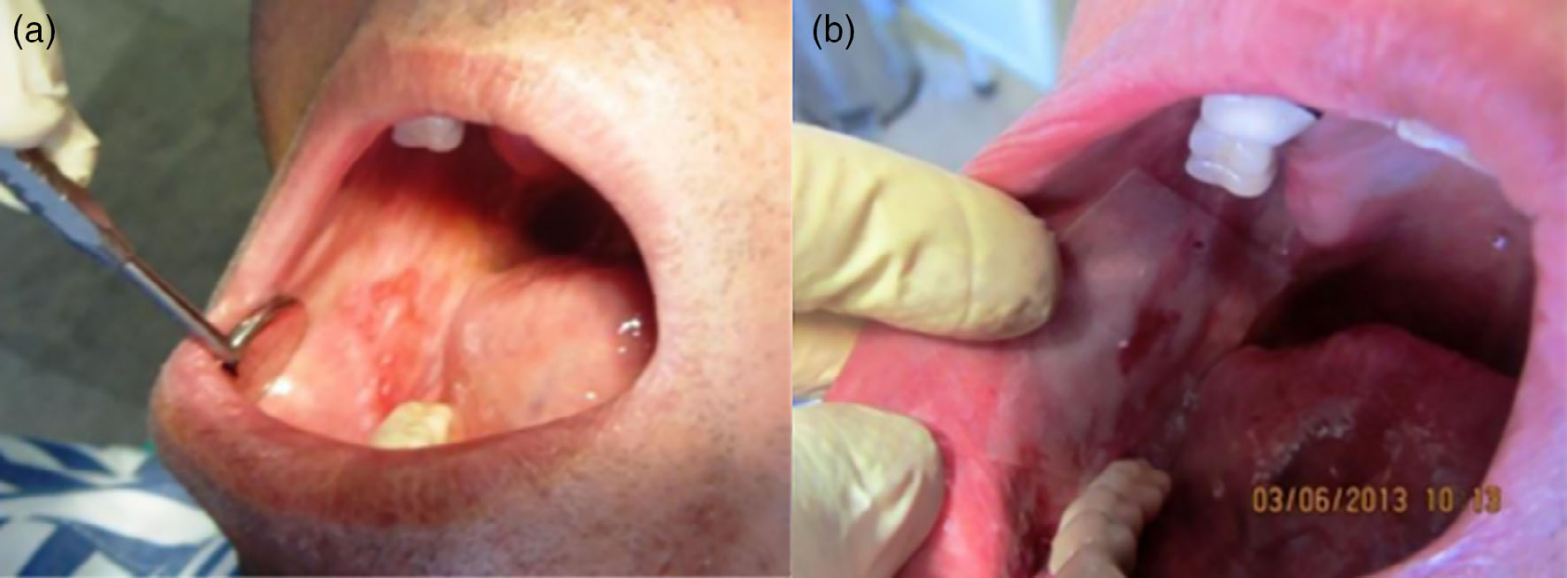
(a) Mouth breathing. (b) Placement of mucoadhesive after 1 min

For participants, a general health check was performed by examiner using a questionnaire, before entering the study. In Group A, the patients were instructed to place 2% MMF mucoadhesive on one of the lesions and a placebo on the other one, twice daily, after breakfast at 10 o'clock and in the afternoon at 5, for 4 weeks. In order to make the study double‐blinded, a third person selected the case and control sites. Neither patients nor researchers were aware of which medication was being administered. In Group B, the patients only used 2% MMF mucoadhesive on the lesion, in the same manner. All the patients were instructed to breathe through the mouth before drug use to make the surface dry and then place the mucoadhesive on the lesion and wait for 1 min for its attachment and then let it dissolve without peeling it off. The patients were asked to avoid eating, drinking and mouth washing for at least 10–15 min (Figure 2).

### 2.4 Clinical assessment

Since burning and pain are close interpretations in symptomatic OLP patients, both severity of burning sensation and pain was measured by VAS scale. This scale is a 10‐cm line, in which 0 indicates no pain and 10 indicates excruciating pain. At baseline and weekly follow‐ups, patients first marked a point on this line according to the severity of pain and VAS scale was measured from 0 to the patient's point in centimeters and then lesion size was measured by a sterile digital caulis in millimeters.

They were also asked about drug compliance and side effect of gastrointestinal system, rash, fever, and headache each week and by phone calls between weekly visits, as all of these were rare side effects reported by systemic use of MMF.

During the study, two patients from Group A and two patients from Group B were excluded because of inability to come for follow‐up visits.

## 3 RESULTS

Twenty‐three patients (17 men and 6 women with the mean age of 45.6 years) completed the 4‐week treatment (Group A: 8 and Group B: 15); At baseline, in Group A, participants reported a median VAS of 6.25 in the case side similar to that reported for the control side which was of 5.52, the lesion diameter was also similar in both sides (Table 1). In both groups, there were no significant differences in lesion size and VAS neither at baseline nor during the Weeks 1–3 of treatment. However, at the end of the treatment (Week 4), the differences became significant in both VAS and lesion size (Tables 1, 2, 3, 4).

**TABLE 1:** Comparison of clinical response of case and control sides in group A

	Baseline		First week		Second week		Third week		Fourth week	
	Case	Control	Case	Control	Case	Control	Case	Control	Case	Control
Lesion size (mm) Group A	28/19	29/63	20/00	25/28	15/80	23/89	21/74	26/43	13/63	23/35
VAS (cm) Group A	6/25	5/52	4/93	5/84	2/76	2/03	2/03	1/13	2/26	2/16

Abbreviation: VAS, visual analogue scale.

**TABLE 2:** Comparison of clinical response in group B

	Baseline	First week	Second week	Third week	Fourth Week
Lesion size (mm) Group B	26/84	24/76	20/02	20/40	12/09
VAS (cm) Group B	4/44	2/89	2/43	1/98	1/12

Abbreviation: VAS, visual analogue scale.

**TABLE 3:** Comparison of symptoms of case and control sides in Group A

Results variable		Comparison of baseline with week 4	
		*t*	*p* Value
Case side	Lesion size	3.568	.004*
	Pain and burning	3.568	.012*
Control side	Lesion size	1.305	.216*
	Pain and burning	2.479	.048*

**TABLE 4:** Comparison of patient symptoms during follow‐up weeks in Group B

	Comparison of baseline with weekly follow‐ups	*P* Value
Lesion size Group B	Week 1	.727
	Week 2	.228
	Week 3	.388
	Week 4	.002
Pain and burning sensation Group B	Week 1	.79
	Week 2	.59
	Week 3	.517
	Week 4	.004

No adverse effect was reported by patients. Only one of patients complained of bitter taste of MMF patch.

## 4 DISCUSSION

This study aimed to evaluate efficacy of mucoadhesive form of MMF in reduction of pain and burning sensation severity and size of ulcerative OLP lesions, because during clinical examinations, these are the most notable symptoms expressed by patients. Although, VAS is a patient‐based symptomatic evaluation and could be unreliable due to different tolerance threshold of the patients; lesion size is a sign of disease progress and the results of our study showed that topical MMF 2% was able to be effective in reducing both of them and improving symptoms and signs of OLP.As of our knowledge, this was the first time that MMF was used topically to treat OLP, so it was not possible to compare the results of this study with similar studies; therefore, the results were compared with rather similar studies.

Beissert et al. (2006, 2007), compared MMF and azathioprine as adjunct therapy to oral methyl prednisone, in two multicentered, randomized, non‐blinded clinical trials on patients with pemphigus vulgaris, pemphigus foliaceus, or bullous pemphigoid. MMF had impressive rates of complete remission (72 and 100%), with no significant differences between treatment arms regarding cumulative corticosteroid doses. Severe or life‐threatening adverse effects were significantly fewer and hepatic toxicity was significantly lower in the MMF treatment group. Cho et al. (2010) conducted a retrospective chart review of adult patients with lichen planopilaris (LPP) treated with MMF and reported that MMF was effective in reducing the signs and symptoms of active LPP in 83% of patients with failed multiple prior treatments after at least 6 months of treatment. Wee, Shirlaw, Challacombe, and Setterfield (2012) carried out a retrospective review of 10 patients with refractory mucous membrane LP, who used MMF; after 12–15 months of treatment, 40% of oral symptoms decreased, and after 21–24 months a 43% reduction of oral lesions was reported. MMF seems to have a delayed effect on all kinds of LP, as seen in this study compatible with previous studies. Wohrlab et al. (2001) used 2% MMF cream in patients with psoriasis and found a significant decrease in redness, inflammation and desquamation of skin lesions of psoriasis and compared to 1% bethametason‐valerate cream, it was equally effective. Dalmau et al. (2007) initially used MMF with cyclosporine in a patient with refractory OLP, then 2 g MMF daily for 2 months and 1.5 g MMF daily for the next 2 months and reported improvements in ulcerative lesions of OLP. Most studies on the efficacy of MMF in the treatment of different diseases have yielded consistent results and contradictory results are rarely reported. For example, Pisoni et al, in a retrospective study, reported transient partial remission in only two out of seven patients with cutaneous lupus erythematosus (CLE) and noted that MMF was not effective in the treatment of refractory CLE but the patients had previously failed to respond to a mean of four different drugs used to treat SLE skin disease. Thus, the patients in the study could be placed at the severe end of skin disease spectrum (Pisoni et al., 2005). The discrepancy in the results can be attributed to these factors: Firstly, we investigated a different autoimmune disease and secondly another form of MMF was used by the patients during the treatment.

In some studies, despite efficacy of MMF, incidence of certain side effects has been reported (Park, 2011), although all the studies are based on systemic MMF not local form of it. In this study, no side effects were reported by the patients.

As topical steroids are first line treatment of OLP, recent placebo controlled trials on different types of steroids like clobetasol propionate 0.05% mixed with 4% hydroxyethyl cellulose gel (Lodi, Manfredi, Mercadante, Murphy, & Carrozzo, 2020), triamcinolone paste and tacrolimus ointment (Lodi et al., 2020) showed significant reduction of clinical signs of OLP against orabase and 4% hydroxyethyl cellulose gel alone (considered as placebo) in symptomatic OLP patients (Lodi et al., 2020). Our results are comparable to these studies, but unlike these studies, the drug effect durability of MMF patch on OLP is not evaluated and needs more investigation.

There were no significant differences in the severity of pain and burning sensation (VAS) and lesion sizes between the case and control groups at baseline. Therefore, the lesions on both sides were similar in this perspective. In Group A, the differences in VAS and lesion size reduction on case sides, got significant only after 4 weeks completed. Therefore, it can be concluded that use of MMF caused lesion size reduction but drug efficacy was coordinated with duration of drug use; and patient compliance seems to be an important factor to get complete response.

On control sides, the only significant difference was related to VAS at baseline and at the end of the study. Therefore, the drugless mucoadhesive is just effective on VAS rather than size of OLP lesions. In comparison of VAS between case and control sides, most of the patients could not differentiate between right and left sides. They failed to provide a definitive answer and admitted that they provided an approximate answer when they were asked to say in which side they had more pain reduction. Though, the results of Group B (before‐after group) shows a significant decrease of both VAS and lesion size.

Mechanism of action of MMF mucoadhesive was not evaluated in this research study. It can be pointed out that MMF is the prodrug of mycophenolic acid (MPA) and it is rapidly and completely hydrolyzed to its parent compound, MPA, by plasma esterase which is present in all the tissues of the body and even within the cells. Therefore, it can be effective locally on oral mucosa. During the study no side effect were reported except the bitter taste. But adding sweeteners can increase saliva flow leading to fast dissolving of the oral mucoadhesive patch. Limited number of patients in two subgroups makes more studies necessary to perform. Dissolution of the adhesive in the mouth and the possibility of reaching it to the control lesion at the other site of the mouth, could cause bias in the results for group A of trial. However, more studies are needed to investigate mechanism of action of MMF mucoadhesive on reducing symptoms of OLP.

## 5 CONCLUSION

Finally, mucoadhesive MMF seems to be an effective drug with minimal side effects in alleviating symptoms of ulcerative OLP.

## CONFLICT OF INTEREST

None.

## AUTHOR CONTRIBUTIONS

All listed authors have made enough contributions to this study. Negin Samiee drafted the manuscript, explained to patients how to use the allocated drugs, and recorded and extracted the data. Javad Shokri took part in the drug production. Masoumeh Mehdipour proposed the initial idea, Ali Taghavi Zenouz supervised the project, prescribed medicine, reviewed and authorized the manuscript.
